# Mixed Mating System Are Regulated by Fecundity in *Shorea curtisii* (Dipterocarpaceae) as Revealed by Comparison under Different Pollen Limited Conditions

**DOI:** 10.1371/journal.pone.0123445

**Published:** 2015-05-04

**Authors:** Naoki Tani, Yoshihiko Tsumura, Keita Fukasawa, Tomoyuki Kado, Yuriko Taguchi, Soon Leong Lee, Chai Ting Lee, Norwati Muhammad, Kaoru Niiyama, Tatsuya Otani, Tsutomu Yagihashi, Hiroyuki Tanouchi, Azizi Ripin, Abdul Rahman Kassim

**Affiliations:** 1 Forestry Division, Japan International Research Center for Agricultural Sciences (JIRCAS), Ohwashi, Tsukuba, Ibaraki, Japan; 2 Department of Forest Genetics, Forestry and Forest Products Research Institute, Matsunosato, Tsukuba, Ibaraki, Japan; 3 Center for Environmental Biology and Ecosystem Studies, National Institute for Environmental Studies, Onogawa, Tsukuba, Ibaraki, Japan; 4 Hayama Center for Advanced Studies, The Graduate University for Advanced Studies, Hayama, Miura-gun, Kanagawa, Japan; 5 Forestry Biotechnology Division, Forest Research Institute Malaysia (FRIM), Kepong, Selangor Darul Ehsan, Malaysia; 6 Bureau of International Partnership, Forestry and Forest Products Research Institute, Matsunosato, Tsukuba, Ibaraki, Japan; 7 Shikoku Research Center, Forestry and Forest Products Research Institute, Asakuranishi-machi, Kochi-shi, Kochi, Japan; 8 Tohoku Research Center, Forestry and Forest Products Research Institute, Shimokuriyagawa, Morioka, Iwate, Japan; 9 Green Forest Resources, Rawang, Selangor Darul Ehsan, Malaysia; 10 Forestry and Environment Division, Forest Research Institute Malaysia (FRIM), Kepong, Selangor Darul Ehsan, Malaysia; University of Massachusetts, UNITED STATES

## Abstract

The maintenance of mixed mating was studied in *Shorea curtisii*, a dominant and widely distributed dipterocarp species in Southeast Asia. Paternity and hierarchical Bayesian analyses were used to estimate the parameters of pollen dispersal kernel, male fecundity and self-pollen affinity. We hypothesized that partial self incompatibility and/or inbreeding depression reduce the number of selfed seeds if the mother trees receive sufficient pollen, whereas reproductive assurance increases the numbers of selfed seeds under low amounts of pollen. Comparison of estimated parameters of self-pollen affinity between high density undisturbed and low density selectively logged forests indicated that self-pollen was selectively excluded from mating in the former, probably due to partial self incompatibility or inbreeding depression until seed maturation. By estimating the self-pollen affinity of each mother tree in both forests, mother trees with higher amount of self-pollen indicated significance of self-pollen affinity with negative estimated value. The exclusion of self-fertilization and/or inbreeding depression during seed maturation occurred in the mother trees with large female fecundity, whereas reproductive assurance increased self-fertilization in the mother trees with lower female fecundity.

## Introduction

Mixed mating, in which hermaphrodite plant species reproduce by self- and cross-fertilization, has been observed in nearly half of the animal-pollinated plant species [1]. Traditionally, the evolution of selfing and outcrossing has been proposed to involve two opposing genetic forces: the transmission advantage of selfing [2] and the disadvantage of inbreeding depression [3]. Hence, Lande and Schemske [4] predicted just two stable endpoints of mating system evolution: predominant outcrossing with strong inbreeding depression and predominant selfing with weak inbreeding depression. However, many theoretical and, to a lesser extent, empirical studies demonstrated that nonrandom embryo abortion (presumably caused by early inbreeding depression) can increase progeny fitness, presumably via a positive relationship between competitive ability of embryos and seedling or adult fitness [5–7]. Recent theoretical and empirical studies have demonstrated evolutionary stability of an intermediate rate of outcrossing under certain conditions [8–11]. Despite the negative effect of inbreeding depression, several theoretical studies have suggested that self-fertilization has been maintained because it offers reproductive assurance [12–15]. And self-fertilized seeds, despite having less viability, have a function to increase population level fitness by acting as seed predator sinks [16], which may be one of the reasons why self-fertilization is maintained at certain level. Pollen limitation, e.g., caused by the lack of mates or pollinators, is one of the main forces driving the evolution of autonomous self-fertilization because autogamous seeds provide reproductive assurance [16–18]. Pollen discounting is the reduction of male reproductive success by outcrossing, that may also lead to increased selfing rate, due to the decrease in the amount of exported pollen [19–23]. However, seeds fertilized by self-mating are exposed to reduction of fitness by inbreeding depression. Thus, the level of mixed mating, in other words stable selfing rates, was regulated by inbreeding depression and pollen limitation (combined with pollen discounting) using a theoretical model [23]. The determinant factors of pollen limitation and discounting are pollen dispersal pattern, male fertility and other ecological factors (ex. stigma clogging), which regulate the mixed mating patterns of plant species, apart from the level of inbreeding depression. We attempted to simultaneously estimate the parameters of pollen dispersal and male fecundity, together with inbreeding depression, for a mixed mating tropical forest species, in order to identify the conditions of maintaining self-fertilization.

Population density is one of the most important factors regulating pollen limitation, insufficient pollen or pollinators are expected to cause positive density dependence in fecundity for self-incompatible plants [24]. Tropical trees typically exhibit positive density-dependent reproduction because most rely on insects for their pollination and are largely outcrossed and self-incompatible [25–28]. Previous studies have shown that low density populations of some trees have fewer pollen donors and reduced outcrossing, providing indirect evidence that density affects reproductive output through pollen limitation [29, 30]. In Southeast Asia, trees of the family Dipterocarpaceae are widely distributed but are dominant in rain forests [31, 32]. Pollination of section *Mutica* in genus *Shorea* (Dipterocarpaceae) has been shown to be density-dependent; lower mature seed production and an increase in selfing rate have been have been reported to be associated with low population density [33, 34]. These findings provide evidence for reproductive assurance. On the other hand, inbreeding depression has been observed to reduce seed mass in a *Shorea* species [33], which may in turn reduce the fitness of selfed seeds. Many studies have shown that dipterocarp species adopt a mixed mating strategy. However, it has been not verified whether the mixed mating of dipterocarps is regulated by the relative amount of self vs. outcross-pollen landing on stigma [competing selfing, 14] or coupled with inbreeding depression. If these factors do play a role in the control of mixed mating system of dipterocarps, it will be interesting to understand the underlying mechanism. Therefore, we revised the modeling of pollen dispersal and variance of male fecundity [35] by incorporating a parameter expressing inbreeding depression until seed maturation. In addition, we extend our study to another population with different population density (selectively logged forest). This modeling and comparative approach between populations with different population density allows us to infer the effect of pollen limitation and inbreeding depression on mixed mating system of the dipterocarp species.

In the present study, we hypothesized that early inbreeding depression (until seed maturation) reduces the number of selfed seeds in undisturbed forests in relation to the content and magnitude of the pollen cloud, which is regulated by individual male fecundity and pollen dispersal patterns. We also predicted pollen cloud conditions that would ensure reproductive assurance and/or reduce self-fertilization, which may contribute to the maintenance of mixed mating system.

## Materials and Methods

### Research plot, sampling strategy and molecular analysis

This research is not related to Human Subject Research and Animal Research. Forest Research Institute Malaysia (FRIM) has permission from authority to manage Semangkok Forest Reserve (Selangor Forest department, Malaysia). Japan International Research Center for Agricultural Sciences was guaranteed to conduct research there by FRIM under the MoU and research agreement between the two institutes. Semangkok Forest Reserve is located in and governed by the Selangor state (2°58'N, 102°18'E), 60 km north of Kuala Lumpur, in the Malaysian Peninsula and is a designated hill dipterocarp forest conservation area. In 1993, Niiyama *et al*. [36] established an undisturbed, 6-ha permanent plot (200 m × 300 m) on a narrow ridge and steep slope, ranging from 340 to 450 m above sea level. Another ca. 4-ha (100 m × 400 m) permanent plot was established within a selectively logged area of forest in 1994 and was extended to about 5.4-ha (ca. 140 m × 400 m) in 2007 [37]. All trees in both plots with a diameter at breast height (dbh) greater than 5 cm were tagged. Selective logging was carried out in part of the reserve in 1988. The felling intensity was 10% of the total basal area (BA) of all tree species larger than 5 cm dbh (total BA was ca. 35 m^2^ ha^-1^ before and 31.5 m^2^ ha^-1^ after the logging). Consequently, a large difference in adult tree density emerged between the two plots (21.3 trees ha^-1^ in the undisturbed and 6.7 trees ha^-1^ in logged plots) in 1998.

**Fig 1:**
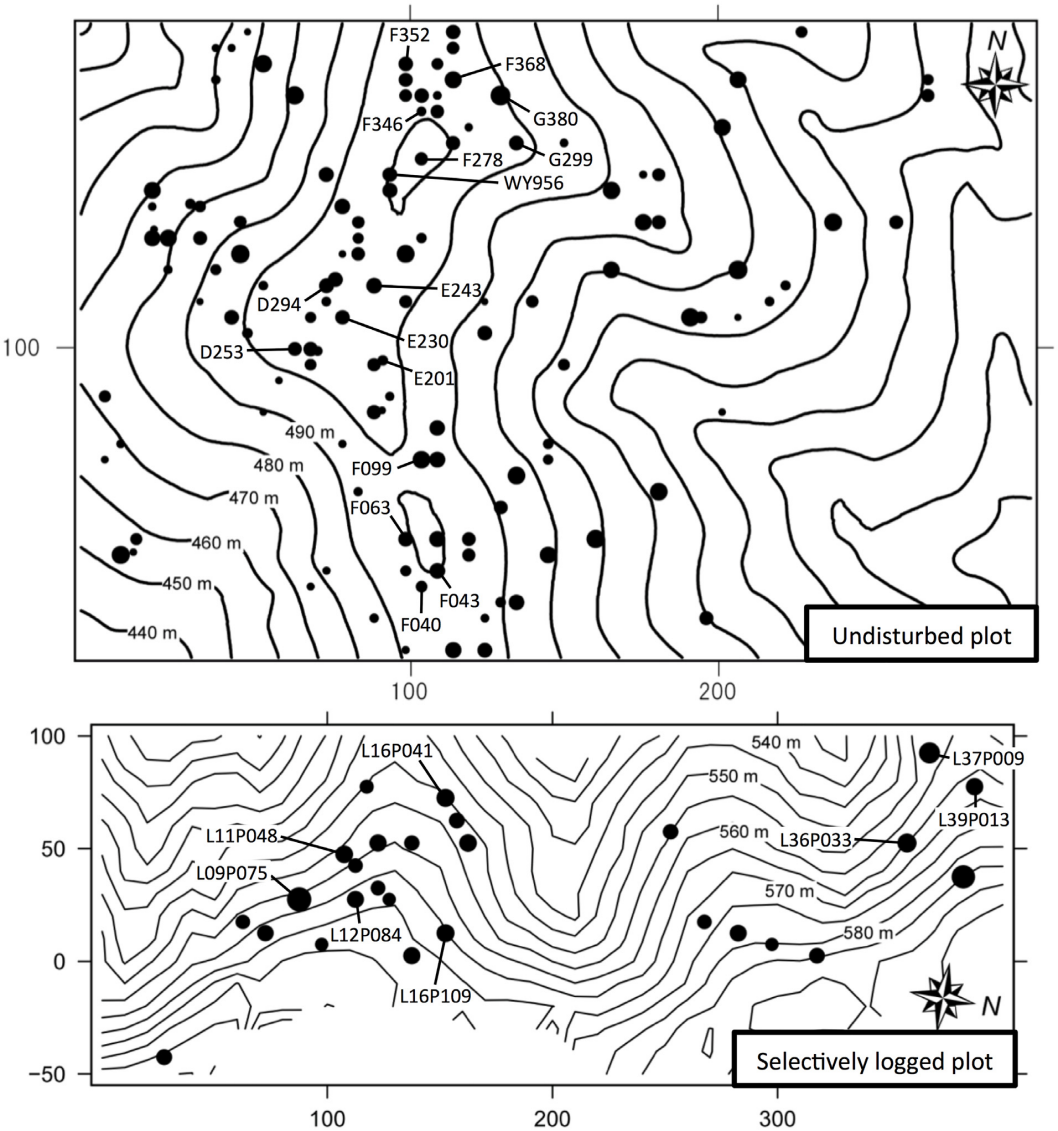
The distribution of adult *Shorea curtisii* trees (with dbh greater than 20 cm) in the 6-ha the undisturbed plot and 5.5-ha selectively logged plot. The black circles and their diameter show the locations and dbh of the adult trees, respectively. The black circles with tree tag no. indicate seed collection trees (mother trees).

Samples of the inner bark or leaves of all *S*. *curtisii* individuals growing within both the plots (17 and three trees were growing in areas of the study plots adjacent to the undisturbed and logged plots, respectively) with dbh greater than 20 cm (defined as adult trees) were collected; these trees were considered to be the candidate pollen donors (Fig 1). Mass and sporadic synchronized flowering events were observed in SFR in 1998, 2005 and 2002, respectively. Seeds were collected from under the canopies of seven (in 1998), ten (in 2005), 11 (in 2002) selected mother trees in the undisturbed plot, and four (in 1998), eight (in 2005) selected mother trees in the logged plot. We could not collect seeds from the logged plot in 2002, because mature seeds were not available in the plot. The number of seeds analyzed from each flowering event is presented in Table 1. Genomic DNA was extracted directly from the embryos of seeds and leaves or inner bark of the potential pollen donors, using a method described by Murray & Thompson [38]. The extracted DNA from the adult trees was further purified using a High Pure PCR Template Preparation Kit (Roche). After RNA digestion, the DNA from the embryo of seeds and the potential pollen donors was diluted to a concentration of 2 ng/μL. DNA extraction was conducted in FRIM.

**Table 1:** Numbers of mother trees and seeds analyzed for paternity analysis, rates of categorical paternity, immigration and selfing revealed by paternity analysis and average and standard deviation of selfing rate of flowering events in the undisturbed and selectively logged plots.

Flowering event and plot	Number of mother trees	Number of seeds for analysis	Rate of immigrant	Rate of seeds sired by pollen donors inside the plot	Average of selfing rate	SD of selfing rate
		*n*	*m*	*1—m*	*s*	
*Undistubed plot*						
1998	8	339	0.2419	0.7581	0.0650	0.0573
2002	11	409	0.2249	0.7751	0.1418	0.2096
2005	10	744	0.3212	0.6788	0.0784	0.0954
Overall	19	1492	0.2768	0.7232	0.0970	0.1418
*Selectively logged plot*						
1998	4	182	0.2912	0.7088	0.5157	0.3314
2005	8	546	0.2912	0.7088	0.5476	0.2416
Overall	12	728	0.2923	0.7088	0.5528	0.2605

All samples were genotyped with ten microsatellite markers previously developed by Ujino *et al*. [39], Lee, Tani [40] and Lee, Ng [41]. Polymerase chain reaction (PCR) amplifications were carried out in total reaction volumes of 10 μL using a GeneAmp 9700 (Applied Biosystems). The PCR mixture contained 0.2 μM of each primer, 1x QIAGEN Multiplex PCR Master Mix (Qiagen), and 0.5–3 ng of template DNA. The temperature profile was as follows: 15 min at 95°C, then 30–35 cycles of 30 sec at 94°C, 90 sec at 50–57°C and 90 sec at 72°C, followed by a 10 min extension step at 72°C. Amplified PCR fragments were electrophoretically separated using a 3100 genetic analyzer (Applied Biosystems) with a calibrated internal size standard (GeneScan ROX 400HD). The genotype of each individual was determined from the resulting electropherograms using the GeneMarker (SoftGenetics). The genotypic data from the undisturbed plot were the same as reported in a previously published paper [35]. The microsatellite genotyping was partly performed in Forestry and Forest Products Research Institute (FFPRI), Japan. DNA materials were transferred from Malaysia with permission by FRIM under the MOU between FRIM and JIRCAS. The genotype data were deposited into Dryad (DOI; http://dx.doi.org/10.5061/dryad.7k434).

### Microsatellite marker diversity, paternity assignment and mating system

Before assigning paternal parents, the offspring with genotypes conflicting with the assumed maternal tree genotypes were excluded from the offspring genotype array. Such conflicts may arise where seeds are collected from under the canopy of a presumed maternal tree because seed dispersal and canopy overlaps between individuals of the same species may result in mixed seed collections. After exclusion of anomalous offspring, we used categorical allocation in combination with an exclusion procedure to identify candidate paternal trees. The paternity of each offspring was determined by likelihood ratios and their confidence levels (greater than 95%) were derived using CERVUS ver. 3.0 [42, 43]. To conduct likelihood tests in CERVUS, we created 100,000 simulated offspring genotypes from 600 potential paternal candidates, with a mistyping rate of 0.1% in the categorical allocation of both plots. However, if the paternal candidates identified by the likelihood procedure had more than two loci mismatches in the simple exclusion procedure, we assumed that the paternal tree of the offspring was located outside the plot and the seed was not assigned to any of the paternal candidates. Electropherograms were double-checked to confirm mismatches between the offspring and paternal candidate in order to minimize genotyping errors. Although the high exclusion power of the microsatellite markers was generally capable of assigning paternity to a single candidate or immigrant pollen, the difference in LOD score between the first and second candidates was not significant for four (1998), ten (2002), six seeds (2005) in the undisturbed plot [35], and for two (1998), four (2005) seeds in the selectively logged plot. As a result, the number of genotyped seeds used for the subsequent analysis was shown in Table 1.

### Modeling pollen dispersal, variance of male fertility and inbreeding depression

Tani, Tsumura [35] have already developed the Bayesian approach to estimate the parameters of both dispersal kernel and male fecundity, which were based on modification of previous procedures [44–47]. Here, we incorporated parameters representing the intensity of early inbreeding depression prior to seed maturation stage into the mating model because we analyzed the matured seeds for paternity analysis.

#### Parameters and mating model

We applied power exponential dispersal kernel to the model based on the probability that pollen travels from its origin (0,0) to position (*x*,*y*) in the pollen cloud [48, 49]. This approach has been used in many previous studies [44, 50, 51] in the form

$$p\left(a,b;r\right)=\frac{b}{2\pi a^{2}\Gamma \left(2/b\right)}\exp\left[-{\left(\frac{r}{a}\right)}^{b}\right]$$

where *Γ* is the classically defined gamma function [52] and

$$r=\sqrt{\left(x^{2}+y^{2}\right)}$$

is the pollination distance. The parameter *b* is a shape parameter, affecting the tail of the dispersal distribution, and *a* is a scaling parameter. See detail in [50, 53]. The male fecundity of each mature tree *j* in the plot was denoted *F*_*j*_ and assumed to follow a log-normal distribution of mean = 1 and variance Σ^2^ [44]. Hence,

$$E\left[F_{j}\right]=1,\mathrm{var}\left[F_{j}\right]=\Sigma^{2}$$

Therefore, the logarithm of mature tree fecundities follows a normal distribution:

$$f_{j}=\ln\left(F_{j}\right),f_{j}∼Norm\left(-\frac{\sigma^{2}}{2},\sigma^{2}\right)$$

where σ^2^ = ln(Σ^2^+1).

The variance of male fecundity is related to the ratio of the observed density of pollen donors (*d*_obs_) to the effective density of pollen donors (*d*_ep_), defined as the number of equifertile pollen donors per unit area, which provides a probability of co-paternity before dispersal equal to that observed [54]. This relationship can be written as follows [44, 51]:

$$\frac{d_{obs}}{d_{ep}}=\frac{\mathrm{var}\left[F_{k}\right]+E{\left[F_{k}\right]}^{2}}{E{\left[F_{k}\right]}^{2}}=\Sigma^{2}+1=e^{\sigma^{2}}$$

#### Probability of paternity assignment

Unlike the classical mating models [55, 56], we categorized the probabilities of paternity of seeds from the *i*th mother tree into two levels: *m*_*i*_, the probability of an unrecorded pollen donor from outside the plot (immigration); and (1-*m*_*i*_), the probability of fertilization by any pollen donor candidates from within the plot (including self-fertilization). Probabilities were obtained directly from the paternity analysis. The probability *m*_*i*_ was obtained from *ñ*_*i*_/*n*_*i*_, where *ñ*_*i*_ is the number of seeds whose paternal donor was not detected in the plot and *n*_*i*_ is the number seeds for paternity analysis from the *i*th mother tree. Subsequently, we included self-fertilized seeds to investigate the effect of early inbreeding depression. We separately used two distances, 0 m and 1 m, for self-pollen travel. The 1 m of self-pollen travel can explain geitonogamous pollen dispersal and is expected to reduce the pollen dispersal kernel’s sensitiveness to dispersal over short distance [53]. The ratio of the *j*th pollen donor candidate’s mating contribution to the *i*th mother tree in relation to the total number of seeds whose pollen donors were identified in the plot was defined as

$${\hat{q}}_{ij}=n_{ij}/\left(n_{i}-{\tilde{n}}_{i}\right),$$

where *n*_*ij*_ is the number of seeds from the *i*th mother tree sired by the *j*th paternal candidate. The expected probability of the *i*th mother tree’s seeds sired by the *j*th pollen donor under outcrossing was modeled as

$$q_{ij}=\frac{\exp\left\{f_{j}+c\times w_{ij}-{\left(\frac{r_{ij}}{a}\right)}^{b}\right\}}{\sum_{k=1}^{N}\exp\left\{f_{k}+c\times w_{ik}-{\left(\frac{r_{ik}}{a}\right)}^{b}\right\}}$$ (model 1)

where *q*_*ij*_ is the expected rate of mother tree’s offspring sired by the *j*th pollen donor. *f*_*j*_ is again the parameter for logarithmic male fecundity of *j*th pollen donor with the assumption of a normal distribution of mean-σ^2^/2 and variance σ^2^ [44]. *a* and *b* were parameters for the dispersal kernel with exponential power distribution [reviewed in 50]. Note that the normalizing constant of the dispersal kernel cancels out and only one or zero candidate father was assigned to each offspring. *c* is the parameter for self-fertilization and 1 was substituted with *w*_*ij*_ when *i*th mother tree is same as *j*th candidate pollen donor, otherwise 0 was substituted, for indexing selfing and outcrossing, respectively. It might be argued that we have not directly measured biological processes. However, if the posterior distribution of *c* is significantly lower than zero in model 1, the self-pollen cloud over the mother tree is underestimated compared to the number of detected selfed seeds, implying that self-pollen is selectively excluded from mating. Biologically, exclusion of self-pollination by various mechanisms of partial self-incompatibility (including exclusion of self pollen as a consequence of pollen competition), and inbreeding depression are involved in the process to exclude self-fertilized seeds. If the posterior distribution of *c* is significantly higher than zero in model 1, the self-pollen cloud over the mother tree is overestimated compared to the number of detected selfed seeds, implying that self-pollen is favored for mating. Biologically, prior and delayed self-fertilization, pollen discounting and outbreeding depression are among the factors favoring self-fertilized seeds. Hereafter, the parameter *c* is called as self-pollen affinity.

The second scenario examined the effects and variance of early inbreeding depression between mother trees using the following model:

$$q_{ij}=\frac{\exp\left\{f_{j}+c_{i}\times w_{ij}-{\left(\frac{r_{ij}}{a}\right)}^{b}\right\}}{\sum_{k=1}^{N}\exp\left\{f_{k}+c_{i}\times w_{ik}-{\left(\frac{r_{ik}}{a}\right)}^{b}\right\}}$$ (model 2)

Here, *c*_*i*_ is a parameter describing the overall effect of self-pollen affinity of the *i*th mother tree assumed to follow a normal distribution of mean *μ*_*s*_ and variance *σ*_*s*_^2^. We applied model 2 only for 2005 event of the logged plot and all the events of the undisturbed plot because we could not obtain enough sampled seeds and mother trees from 1998 event of the logged plot.

The conditional likelihood function for *M* mother trees was expressed as

$$L\left(q|F,a,b,c,d\right)=\prod_{i=1}^{M}\prod_{j=1}^{N}q_{ij}^{n_{ij}}$$

The full posterior distribution for model 1 was as follows:

$$p\left(F,\sigma_{f}^{2},a,b,c|q\right)=L\left(q|F,a,b,c\right)p\left(f|\sigma_{f}^{2}\right)p\left(a\right)p\left(b\right)p\left(c\right)h\left(\sigma_{f}^{2}\right)$$

and for model 2 was as follows:

$$p\left(F,\sigma_{f}^{2},a,b,c,\mu_{s},\sigma_{s}^{2}|q\right)=L\left(q|F,a,b,c\right)p\left(f|\sigma_{f}^{2}\right)p\left(a\right)p\left(b\right)p\left(c|\mu_{s},\sigma_{s}^{2}\right)h\left(\sigma_{f}^{2}\right)h\left(\mu_{s},\sigma_{s}^{2}\right)$$

The prior parameter values of *F*_*j*_ were assumed to be independent and have the same prior distribution. The log-scale male fertility, *p*(*f*|σ_f_^2^), had a normal distribution with mean—*σ*_*f*_^2^/2 and variance *σ*_*f*_^2^ [52]. The inverse of the hyper-parameter (*σ*_*f*_^2^) was assumed to follow a gamma distribution with values of 0.001 for the shape and 0.001 for the rate parameters, as this represents little *a priori* information. For the mutually independent dispersal kernel parameters, *a* and *b*, we also assumed a prior gamma distribution of (0.001, 0.001) for all models. In model 1, the parameter *c* was assumed to have normal distribution with a mean of 0 and precision of 0.0001 as prior. In model 2, two hyper parameters of mean *μ*_*s*_ and the inverse of variance σ_s_^2^ of *c*_*i*_ were assumed to follow normal (0, 0.001) and gamma (0.001, 0.001) prior distributions, respectively.

#### Bayesian estimation using the Markov Chain Monte Carlo (MCMC) algorithm

The Bayesian analysis calculates the posterior distributions of the parameters, producing conditional distributions that are updated on the basis of observations [57]. Prior distributions were first defined and then modified according to observations concerning the probability of the paternal origin of the seeds. Re-parameterization to fit the data was performed by MCMC sampling using the JAGS software on the R platform [58]. The convergence of MCMC was assessed on the basis of observations after every nine iterations, according to the behavior of three chains with respect to all estimated parameters; these were visualized using CODA, (Convergence Diagnostic and Output Analysis) [59]. The value of Gelman and Rubin’s convergence diagnostic was estimated to validate the convergence of MCMC for each parameter [60].

#### Magnitude and composition of self and outcross pollen in pollen cloud

Given the parameter values of each MCMC iteration in model 2 for male fecundity (*F* = {*F*_*j*_}_*j = 1…N*_) and the dispersal kernel *p*(*a*,*b*; *x*,*y*), *j*th tree’s male fecundity in *i*th mother’s pollen cloud (*π*_*ij*_) and self-pollen in the pollen cloud of *i*th mother (*π*.*self*_*i*_) was calculated from

$$\pi_{ij}=F_{j}\times p_{ij}\;$$

and

$$\pi .self_{i}=F.ith\_mother_{j}\times p_{ij},\;$$

respectively, where *p*_*ij*_ = *p*(*a*,*b*;*r*_*ij*_). Here, *F*.*ith_mother*_*j*_ is the male fecundity of the *j*th pollen donor, which is equivalent to the *i*th mother tree. On the other hand, the total amount of outcross-pollen was calculated by subtracting the amount of self-pollen from the pollen cloud size of the *i*th mother tree:

$$\pi .out_{i}=\sum_{j=1}^{N}\pi_{ij}-\pi .self_{i}$$

The proportion of self-pollen to pollen cloud on the *i*th mother tree was calculated from

$$\rho_{i}=\pi .self_{i}/\sum_{j=1}^{N}\pi_{ij}$$

In order to evaluate the statistics, the 95% and 50% credibility intervals and median were sampled from the distributions that are output from each MCMC iteration.

## Results

### Mating system

The ten microsatellite markers showed sufficient variation to conduct a paternity analysis of each seed collected in both the undisturbed plot and the logged plot. On average, 12.3 and 8.4 alleles, ranging from 5–17 and 4–13 alleles per locus, were detected in the *S*. *curtisii* adult tree samples from the undisturbed and selectively logged plots, respectively. High polymorphic levels were detected in both plots (0.691 and 0.657 of PIC (Polymorphic Information Contents) values in the undisturbed plot and the logged plot, respectively), which were associated with the markers, resulting in a high total exclusion probability for identifying the second parents of offspring in the paternity analyses (0.999756 and 0.999379 in the undisturbed plot and the logged plot, respectively). Thus, paternity was assigned with high resolution (S1 Table).

The *m*_*i*_ pollen rates were virtually same as the rate of pollen dispersal from outside the plot (immigrant) because the high paternity exclusionary power of the microsatellite markers reduced the rate of cryptic pollen dispersal to a negligible level. The detected rates of pollen dispersal from outside the plot fluctuated between mother trees. The majority of mother trees produced relatively fewer seeds due to fertilization by immigrant pollen (less than 30% on average) during both events and in both plots. The proposed models require information on the paternity of seeds sired by all potential paternal trees inside the plot (including each mother tree). Therefore, approximately 70% of the collected seeds, 1-*m*_*i*_ from both plots and the three events were considered informative for modeling (Table 1). On the other hand, the estimated selfing rates (*s*) according to the paternity analysis showed a distinct difference between the two plots. Although a low selfing rate was detected for all mother trees in the undisturbed plot, more than 50% of seeds from mother trees in the logged plot were selfed and the selfing rate clearly fluctuated among the mother trees (Table 1).

### Comparison of estimated parameters for inbreeding depression between the sites (model 1)

Gelman and Robin’s convergence diagnostics showed that the posterior distributions of parameters generated by MCMC did not converge for the logged plot in 1998 event. This may be due to the small number of mother trees. However, the analyses for the undisturbed plot at all events and the logged plot in 2005 gave values of nearly 1.00 for the diagnostic of each parameter, although the parameters for the dispersal kernel gave slightly higher values for the selective logged plot in 2005 and undisturbed plot in 1998 (Table 2). These good convergences are probably due to the larger size of mother trees (Table 1). We concluded that the posterior distributions predicted by the latter four analyses should be reliable. When the posterior distribution of each parameter was compared between two definitions of distance of self-pollen travel (0 m or 1 m), significance level of parameter *c* was not changed although the posterior estimation was different between the definitions (Table 2 and S3 Table). We, therefore, henceforth applied the posterior distributions from the definition of 1 m as self-pollen travel. The 95% posterior credibility intervals of parameter *c* estimated for both events in the logged plot crossed through zero even though the analysis for 1998 did not converge (Table 2). However, the three events in the undisturbed plot were associated with negative ranges for the 95% posterior credibility intervals of parameter *c* (Table 2). This suggests that self-pollen was excluded until seed maturation in the undisturbed plots but not in the logged plot.

**Table 2:** Posterior median and 95% (50%) Bayesian Credibility of parameters for dispersal kernel, male fecundity variation and self pollen affinity from the model 1 when 1 m was defined as self-pollen travel.

Parameter	2.50%	25%	50%	75%	97.50%	*R* ^1)^
*Selectively logged plot in 1998*						
*a*	4.158E+246	43.898	0.036	0.006	0.001	3.16
*b*	1.079E-93	0.156	0.192	0.234	0.649	2.02
*c*	-1.310	0.310	1.294	3.112	5.495	3.74
*σ*_*f*_	0.866	1.256	1.527	1.863	2.786	1.00
*d*_*obs*_/*d*_*ep*_	2.118	4.840	10.308	32.102	2350.429	-
*Selectively logged plot in 2005*						
*a*	26.849	3.240	0.543	0.085	0.006	1.38
*b*	0.207	0.260	0.316	0.411	0.682	1.04
*c*	-0.849	-0.113	0.337	0.843	1.806	1.04
*σ*_*f*_	1.084	1.424	1.653	1.928	2.622	1.00
*d*_*obs*_/*d*_*ep*_	3.240	7.592	15.377	41.117	967.156	-
*Undisturbed plot in 1998*						
*a*	18.648	8.300	4.191	1.432	0.140	1.25
*b*	0.282	0.389	0.484	0.580	0.774	1.02
*c*	-1.814	-1.223	-0.894	-0.578	-0.026	1.01
*σ*_*f*_	1.180	1.394	1.522	1.664	1.979	1.00
*d*_*obs*_/*d*_*ep*_	4.028	6.982	10.149	15.941	50.277	-
*Undisturbed plot in 2002*						
*a*	2.713	0.309	0.075	0.017	0.002	1.14
*b*	0.204	0.238	0.272	0.319	0.445	1.03
*c*	-3.138	-2.659	-2.390	-2.103	-1.504	1.02
*σ*_*f*_	1.494	1.732	1.874	2.032	2.392	1.00
*d*_*obs*_/*d*_*ep*_	9.324	20.055	33.561	62.241	304.975	-
*Undisturbed plot in 2005*						
*a*	17.086	9.515	6.380	3.901	1.183	1.00
*b*	0.391	0.489	0.550	0.613	0.747	1.00
*c*	-1.561	-1.094	-0.858	-0.629	-0.213	1.00
*σ*_*f*_	1.301	1.478	1.586	1.703	1.957	1.00
*d*_*obs*_/*d*_*ep*_	5.429	8.899	12.364	18.179	46.074	-

^1)^ Gelman and Rubin’s convergence diagnostic

### Fluctuation of parameters on inbreeding depression among mother trees (model 2)

Model 2 enabled the posterior distribution of parameter *c* to be estimated for each mother tree. When the 95% Bayesian credibility interval of parameter *c*_*i*_ was compared between estimations using 0 m and 1 m as self-pollen travel, three mother trees in the logged plot in 2005 and two mother trees in the undisturbed plot in 2002 represented that the 95% Bayesian credibility interval of parameter *c*_*i*_ was shifted to positive side in the 1 m definition as self-pollen travel. However, most of mother trees showed the same significance level between the two definitions (Table 3 and S3 Table). Therefore, we applied the 1 m definition as self-pollen travel for the interpretation of the result. In the logged plot, the posterior 95% credibility interval of the parameter *c*_*i*_ crossed over 0 or positive range for all the mother trees. Although the parameter *c* significantly deviated from zero to negative values in the model 1 for three events in the undisturbed plot, the posterior credibility intervals of *c*_*i*_ of six mother trees in 1998, of three in 2002 and of seven mother trees in 2005 crossed over zero. The intervals for other mother trees were negative (Table 3). The posterior distribution of parameter *c*_*i*_ fluctuated less in the undisturbed plot than in the logged plot, that was supported by less median of posterior distribution of parameter *σ*_*s*_ of three events in the undisturbed plot.

**Table 3:** Posterior median and 95% (50%) Bayesian Credibility of parameter for self pollen affinity to each mother tree and its variation estimated from model 2 when 1 m was defined as self-pollen travel.

Parameter	Mother tree ID	2.50%	25%	50%	75%	97.50%	*R* ^1)^
*Selectively logged plot in 2005*							
*c*_*1*_	L09P075	-0.455	0.732	1.420	2.145	3.473	1.01
*c*_*2*_	L11P048	0.451	1.569	2.222	2.906	4.197	1.01
*c*_*3*_	L12P084	1.804	3.062	3.811	4.602	6.184	1.00
*c*_*4*_	L16P041	-0.088	1.190	1.937	2.736	4.212	1.01
*c*_*5*_	L16P109	0.675	1.899	2.628	3.411	4.869	1.01
*c*_*6*_	L36P033	-0.849	0.174	0.820	1.544	2.762	1.01
*c*_*7*_	L37P009	-1.890	-0.964	-0.396	0.201	1.222	1.01
*c*_*8*_	L39P013	-2.796	-1.826	-1.247	-0.645	0.399	1.01
*μ*_*s*_	-	-0.583	0.701	1.401	2.143	3.564	1.01
*σ*_*s*_	-	1.060	1.506	1.847	2.299	3.732	1.00
*Undisturbed plot in 1998*							
*c*_*1*_	D253	-1.904	-0.951	-0.465	0.030	0.979	1.01
*c*_*2*_	D294	-1.808	-0.943	-0.499	-0.075	0.719	1.01
*c*_*3*_	E201	-1.599	-0.547	0.015	0.588	1.718	1.00
*c*_*4*_	E230	-10.086	-4.507	-3.037	-2.001	-0.635	1.00
*c*_*5*_	E243	-10.348	-4.895	-3.510	-2.489	-1.006	1.00
*c*_*6*_	F099	-3.644	-1.700	-0.882	-0.107	1.404	1.00
*c*_*7*_	F352	-1.315	0.014	0.833	1.723	3.606	1.00
*c*_*8*_	WY956	-9.175	-3.363	-1.813	-0.708	1.225	1.00
*μ*_*s*_	-	-4.437	-1.947	-1.213	-0.594	0.711	1.00
*σ*_*s*_	-	1.187	1.403	1.533	1.678	2.005	1.00
*Undisturbed plot in 2002*							
*c*_*1*_	D390	-4.421	-3.493	-3.027	-2.563	-1.708	1.01
*c*_*2*_	D511	-0.503	0.409	0.922	1.443	2.516	1.01
*c*_*3*_	E201	-3.724	-2.354	-1.667	-0.969	0.408	1.01
*c*_*4*_	E243	-5.247	-3.598	-2.856	-2.169	-0.914	1.00
*c*_*5*_	E311	-4.253	-2.996	-2.415	-1.862	-0.866	1.01
*c*_*6*_	F043	-7.102	-4.118	-2.932	-1.841	0.239	1.00
*c*_*7*_	F109	-3.137	-2.392	-1.983	-1.566	-0.757	1.03
*c*_*8*_	G097	-5.379	-4.191	-3.640	-3.116	-2.177	1.01
*c*_*9*_	G299	-7.749	-4.941	-3.917	-3.064	-1.661	1.00
*c*_*10*_	G380	-5.743	-4.559	-3.996	-3.472	-2.512	1.01
*c*_*11*_	I242	-4.801	-3.781	-3.253	-2.727	-1.741	1.01
*μ*_*s*_	-	-4.244	-3.138	-2.640	-2.149	-1.172	1.01
*σ*_*s*_	-	0.974	1.400	1.714	2.117	3.329	1.00
*Undistubed plot in 2005*							
*c*_*1*_	D253	-1.648	-0.880	-0.477	-0.083	0.679	1.00
*c*_*2*_	D294	-1.861	-1.102	-0.718	-0.334	0.400	1.00
*c*_*3*_	F040	-3.538	-2.041	-1.388	-0.785	0.314	1.00
*c*_*4*_	F043	-4.811	-2.357	-1.412	-0.578	0.937	1.00
*c*_*5*_	F063	-0.646	0.196	0.642	1.088	1.944	1.00
*c*_*6*_	F278	-1.628	-0.626	-0.110	0.411	1.433	1.00
*c*_*7*_	F346	-0.393	0.435	0.860	1.279	2.079	1.00
*c*_*8*_	F368	-4.724	-3.336	-2.747	-2.222	-1.366	1.00
*c*_*9*_	G299	-3.258	-2.082	-1.532	-1.010	-0.060	1.00
*c*_*10*_	G380	-3.258	-2.291	-1.830	-1.400	-0.631	1.00
*μ*_*s*_	-	-2.231	-1.301	-0.884	-0.483	0.306	1.00
*σ*_*s*_	-	0.710	1.106	1.387	1.747	2.835	1.00

^1)^ Gelman and Rubin’s convergence diagnostic

**Fig 2:**
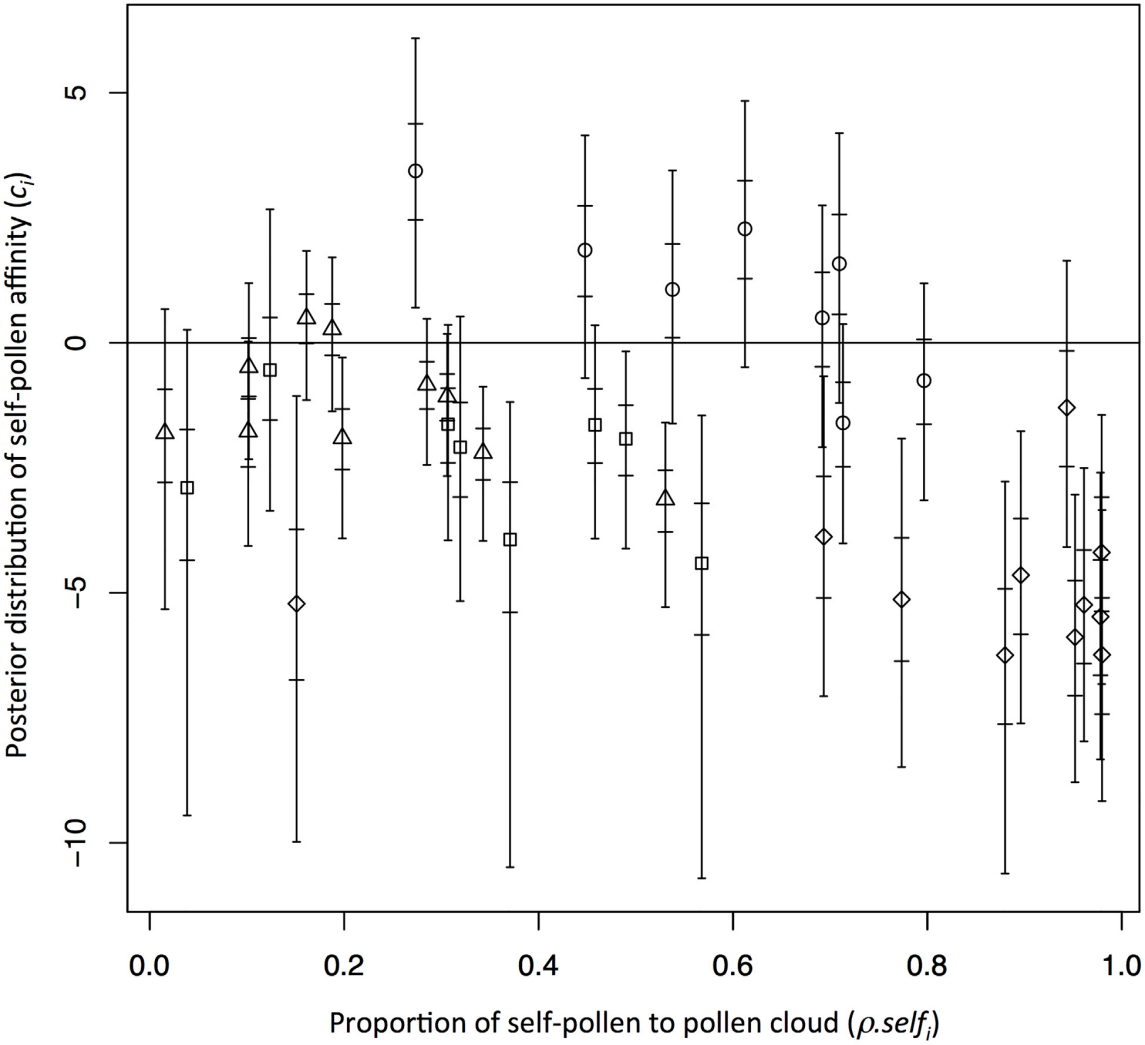
Relationship between the proportion of self-pollen (*ρ*.*self*_*i*_) and self-pollen affinity (*c*_*i*_) of each mother tree. Open circles, open squares, open lozenges and open triangles indicate estimates from 2005 flowering event in the logged plot, and 1998, 2002, and 2005 flowering events in the undisturbed plot, respectively. The long and short bars in each plot represent the Bayesian credibility intervals at 95% and 50% levels, respectively.

**Fig 3:**
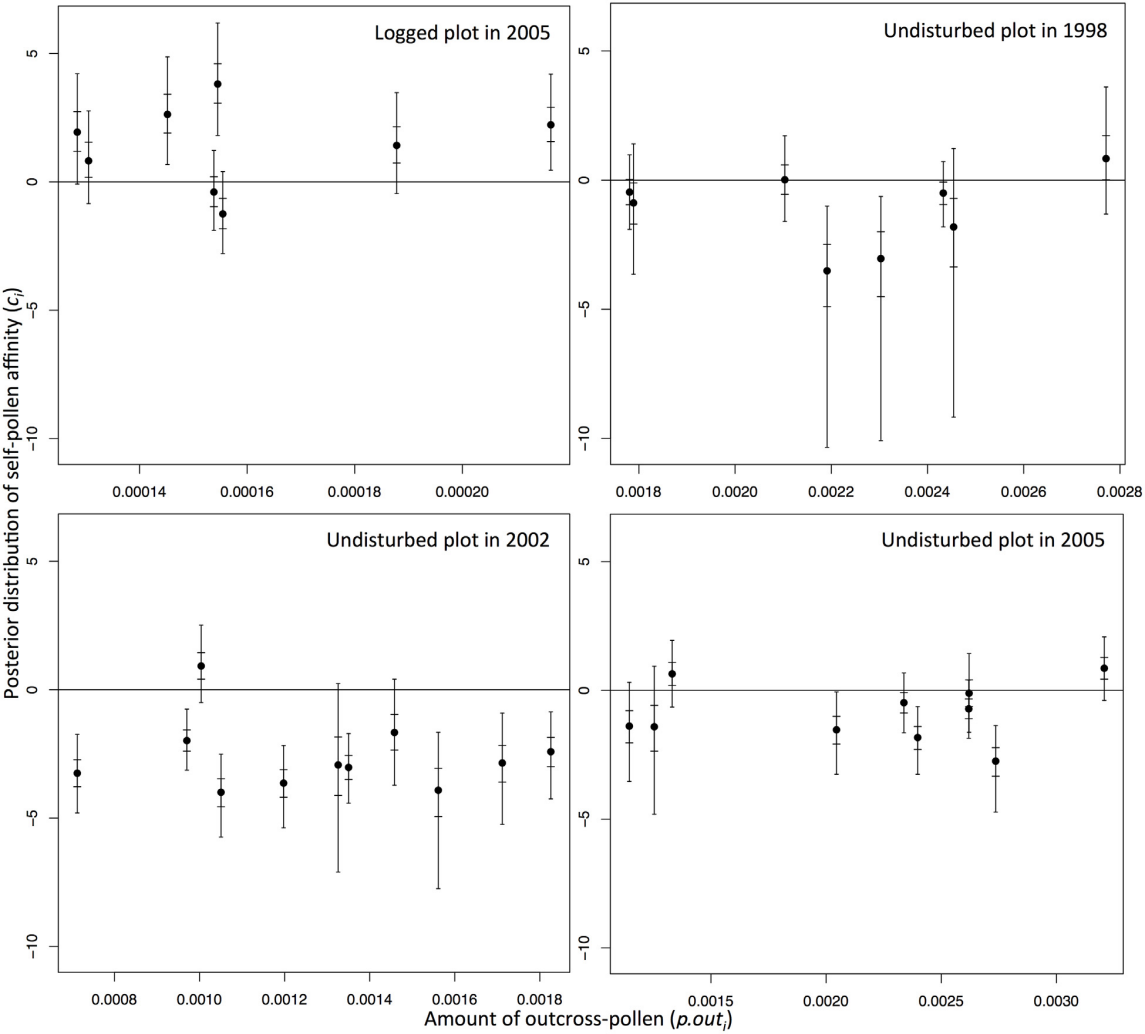
Relationship between the amount of outcross-pollen (*π*.*out*_*i*_) and self-pollen affinity (*c*_*i*_) of each mother tree. The long and short bars in each plot represent the Bayesian credibility intervals at 95% and 50% levels, respectively.

**Fig 4:**
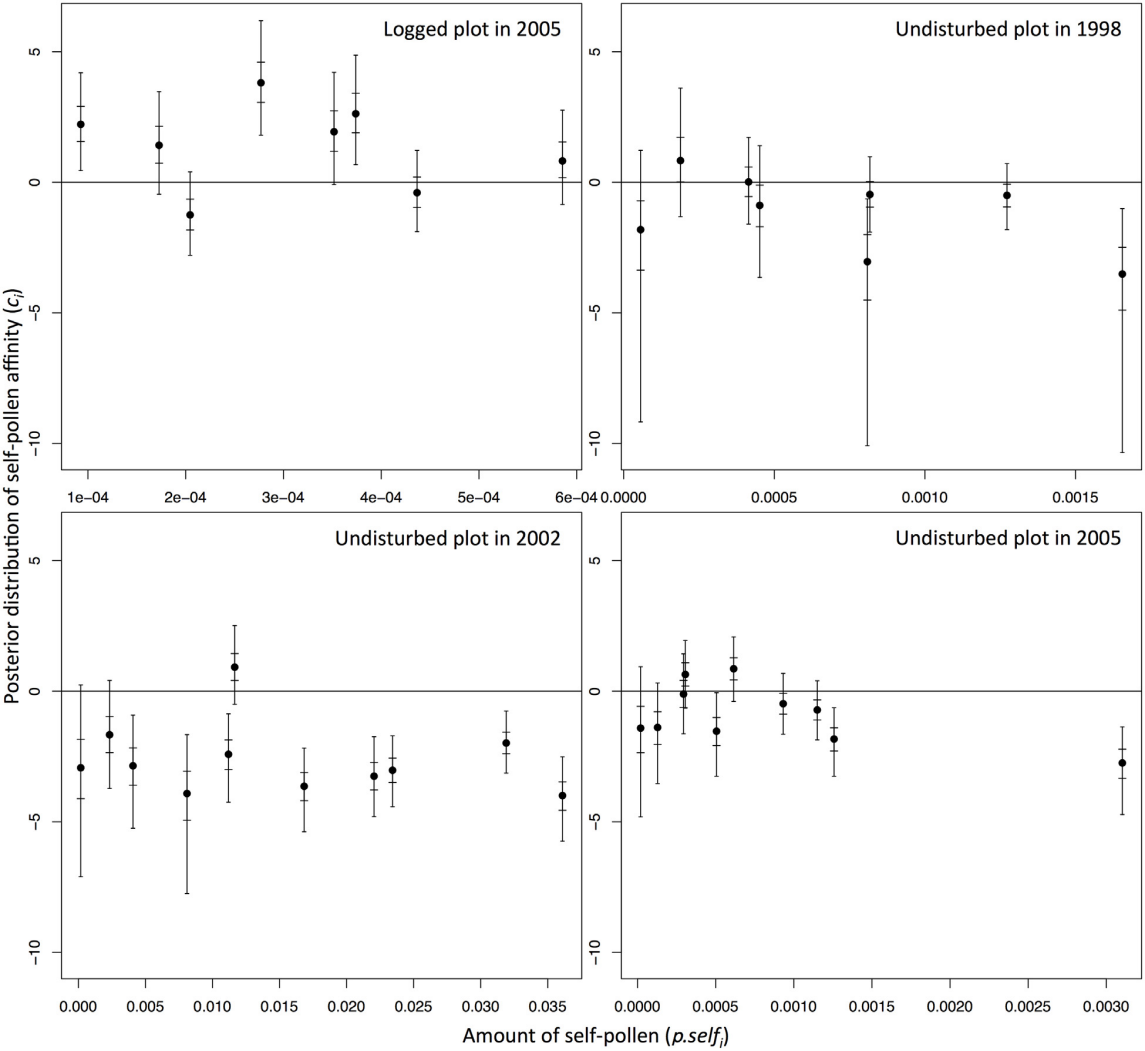
Relationship between the amount of self-pollen (*π*.*self*_*i*_) and self-pollen affinity (*c*_*i*_) of each mother tree. The long and short bars in each plot represent the Bayesian credibility intervals at 95% and 50% levels, respectively.

In an attempt to model the unknown conditions regulating the affinity of self-pollen of the *i*th mother tree (*c*_*i*_), the amounts of self pollen (*π*.*self*_*i*_) and outcross pollen (*π*.*out*_*i*_) of the *i*th mother tree’s pollen cloud and the proportion of self-pollen to pollen cloud of the *i*th mother tree (*ρ*_*i*_) were calculated from the posterior distributions of parameters of male fecundity and the dispersal kernel. The proportion of self-pollen to pollen cloud (*ρ*_*i*_) didn’t show any relationship with the self-pollen affinity (Fig 2). The amount of outcross-pollen also did not show any explicit relationship with the negative significance of self-pollen affinity (*c*_*i*_). For example, mother trees with moderate amount of outcross-pollen in the undisturbed plot of 1998 and 2005 events showed negative significance of self-pollen affinity (Fig 3). On the other hand, mother trees with larger amount of self-pollen (*π*.*self*_*i*_) indicated negative significance of self-pollen affinity in the undisturbed plot (note that no negative significance was observed in the mother trees in the logged plot; Fig 4). However, non-negative significance of self-pollen affinity was detected in two (1998), one (2002) and three (2005) mother trees that showed larger amount of self-pollen than the mother tree showing negative significance, with the smallest amount of self-pollen (Fig 4).

## Discussion

### Reproductive assurance through compensation by selfing

In order to explain reproductive assurance under limited pollen conditions, as observed in the logged plot, we compared parameter *c* in model 1 between the undisturbed and logged plots. The posterior credibility intervals of *c* for the logged plot in both flowering events crossed over zero. These results imply that fertilization of the ovules of mother trees with self-pollen was not restricted and self-fertilized seeds grew into maturity in the logged plot. On the contrary, those from the undisturbed plot in the three events were negative (Table 2). Significant deviation of the posterior probability of *c* from zero to negative values for the undisturbed plot indicates that fertilization with self-pollen was prevented and/or self-fertilized ovules have been aborted before maturation. Several studies [12, 14] have suggested that prior and delayed selfing provides reproductive assurance. On the contrary, in addition to the lack of evidence of the prior and delayed selfing in dipterocarps, our empirical results could be explained by prior and posterior mechanisms of mating under competing selfing. Kenta, Shimizu [61] observed adhesion, germination and growth of self- and outcross-pollen of *Dipterpcarpus tempehes*; the proportion of outcross-pollen tubes that entered the style was 1.7–2.3 times higher than that of self-pollen tubes, although the adhesion and the germination of self-pollen and the growth of self-pollen tubes were not inhibited. This is one of the evidences of prior mechanisms to exclude self-pollen. It has been reported that selfed seeds of dipterocarps generally suffer from inbreeding depression [33, 62], which is one of the posterior mechanisms. Ghazoul, Liston [63] also observed differences in the pollen loads on stigma and fruit development of *S*. *siamensis* between undisturbed and highly disturbed forests and inferred that fertilized inbred fruit was aborted at a very early stage of development. The reproductive assurance observed in the logged plot may arise from competing selfing in this species. In other words, higher outcrossing in the undisturbed plot may be due to the exclusion of large amounts of self-pollen. Nevertheless, it should be noted that the actual pollen dispersal pattern and pollen cloud might be more complicated than the ideal case as suggested in the model. Having said that, the estimated pollen dispersal kernels in the undisturbed plot showed that ca. 20% of the pollen was dispersed up to 3 m from the origin, compared to ca. 70% in the logged plot. Because a 3 m radius is considerably smaller than the size of a typical *S*. *curtisii* crown, large amount of self-pollen is likely to reach the style of each mother tree’s flower (including via auto- and geitono-pollination) in the logged plot. The former and latter observations imply that partial self-incompatibility and inbreeding depression are in operation during seed maturation. Although parameter *c* cannot discriminate these factors, these mechanisms may be important under the conditions where large amount of outcross pollen is available, such as the case of high density population like the undisturbed plot, and can significantly reduce the estimates of parameter *c*.

### Factors that regulate reproductive assurance and maintain outcrossing

No clear relationship between the proportion of self-pollen to pollen cloud (*ρ*.*self*_*i*_) and the posterior median (and 95% Bayesian credibility interval) of self-pollen affinity (*c*_*i*_) was discerned (Fig 2) and the proportion of self-pollen to pollen cloud (*ρ*.*self*_*i*_) also did not indicate clear relationship with the selfing rate (figure not shown, data was shown in S2 Table). These results implied that pollen cloud composition did not have direct influence on the mating system and the genetic composition of matured seeds. Rather, it was the biological factors that control the genetic composition of matured seeds during pre- and post-mating processes.

Caution should be exercised while inferring the levels of reproductive assurance and exclusion of self-pollen because of the large Bayesian credibility intervals of the self-pollen affinity (*c*_*i*_), amount of pollen (*π*_*i*_) and pollen cloud composition (*ρ*_*i*_). This may be due to the calculation procedure, i.e., composition of the pollen cloud was calculated from the posterior distribution of dispersal kernel parameters (*a* and *b*) and male fecundities of all individuals in each plot (*F*_*j*_). This estimated pollen cloud did not take into account the effects of factors such as flowering phenology differences and pollinator behavior on the actual pollen cloud composition. For example, the amount of outcross-pollen may have been over-estimated if the phenology of a mother tree deviated from its neighbors. Despite these limitations, the amount of self-pollen showed relationships with the self-pollen affinity (Fig 4). In contrast, the amount of outcross-pollen did not correlate with the self-pollen affinity (Fig 3). This implies that reproductive assurance is effective when the supply of self-pollen is relatively less (Fig 4). Consequently, the number of selfed seeds is increased. On the other hand, self-pollen was selectively excluded due to partial self-incompatibility and/or inbreeding depression when large amount of self-pollen was available. Thus, once the amount of self-pollen falls below a critical value, the self-pollen affinity and selfing rate increases, maintaining reproductive assurance in *S*. *curtisii*.

Selective abortion of selfed seeds [64–66] may be one of the main factors behind this phenomenon. Because of the short dispersal distance of self-pollen, the amount of self-pollen is nearly corresponding to male fecundity of the mother trees even in the applying 1 m definition of self-pollen travel. Further, the male fecundity should be positively related to the number of female floral organs on each mother tree due to the hermaphroditic nature of the species. The limited resource of mother trees is likely to promote selective abortion of selfed seeds when the mother trees have larger number of female organs. In contrast, mother trees with smaller number of female organs may invest their resources in selfed seeds to ensure reproductive assurance. Naito, Kanzaki [67] estimated flower and seed production of 15 focal trees of *S*. *accuminata* (belonging to same section *Mutica*). All the trees with large flower production showed high rate of premature seed abortion. On the other hand, some trees with smaller flower production showed lower abortion rate (Table 2) [67]. This observation corroborated with our results. The reproductive assurance was related to the level of selective abortion that was possibly equivalent as inbreeding depression represented by the level of parameter *c*_*i*_ in terms of the concept of parameter in this study.

## Supporting Information

S1 Table Genetic diversity and exclusion indices for adult trees (with a dbh larger than 20 cm) of *Shorea curtisii* growing in the Semangkok 6-ha undisturbed and 5.5-ha selectively logged plots. (XLSX)

S2 Table Number of seeds analyzed for the modeling, rates of categorical paternity, immigration and selfing revealed by paternity analysis for *i*th mother tree. (XLSX)

S3 Table Posterior median and 95% and 50% Bayesian credibility of parameters of self pollen affinity from Model 1 and Model 2 when 0 m was defined as self pollen travel. (XLSX)

S4 Table Posterior median and 95% and 50% Bayesian credibility of three statistics on amounts and composition of pollen cloud calculated from the posterior distribution of male fecundity and pollen dispersal kernel for each mother tree derived from model 2 when 1 m was defined as self-pollen travel. (XLSX)
